# American Dental Association

**Published:** 1896-01

**Authors:** 


### Reports of Society Meetings.

### AMERICAN DENTAL ASSOCIATION.

(Continued from Volume XVI., page 690.)

*August 7, 1895.—Second Day.—Evening Session.*

THE meeting was called to order by Dr. Watkins, who presided, and the minutes of the previous session were read and approved.

Dr. Boice offered a resolution that the treasurer be instructed to get a new set of books; also a treasurer's individual badge.

*Dr. Abbott.*—I would like to know what that badge is intended for; for the treasurer or for every individual who pays his dues?

*Dr. Boice.*—It is not a badge for the treasurer alone. It is a badge which is given to every gentleman when he pays his dues.

After some discussion the motion was laid on the table.

The chairman of the Committee on the World's Columbian Dental Congress stated that the proceedings which have been sent to the members is the only report that he has to make. The most important report is the one which Dr. Marshall will make as treasurer of the World's Columbian Dental Congress.

*Dr. Marshall.*—I have prepared, for the benefit of the Association, and also for publication in the journals, a report of the financial condition of the Congress, its receipts and disbursements. In that report I have arranged to have each State and each society get credit for the amount of money which it subscribed before the sessions of the Congress, and also what we received from the memberships.

The report was accepted.

Dr. Crouse moved that the consent of this Association be granted the secretary to cast a unanimous ballot for Dr. Lyman C. Bryan, United States vice-consul to Switzerland, as an honorary member of this society.

Motion carried.

In the absence of Dr. Crenshaw, Dr. Fillebrown reported on behalf of the committee appointed on the President's Address, as follows:

The committee beg to submit the following:

1. That the American Dental Association adopt the suggestion contained in the address, looking to the erection of a suitable monument to the memory of Horace Wells in recognition of his bequest to our profession, and also to incorporate the addresses of Drs. Fillebrown and Garretson in the published transactions of this meeting.

2. We recommend a continuation of the committee appointed to meet and discuss with a similar committee from the Southern Dental Association the feasibility of bringing together the American and Southern Associations in one body.

3. We recommend a further and more persistent effort throughout the society to the suggestion in the address for the appointment of dentists in the army and navy.

4. We would also ask the appointment of a well-selected committee to report twelve months hence upon the suggestion of the address with reference to the condition of the mouth and teeth, and the bearing this must have in the matter of obtaining life insurance.

5. Your committee regard the suggestion of the paper touching the education of our children as one of great interest and importance, and suggest a further discussion and expression of opinion from this body upon the question.

6. We recommend the suggestions of the address to medical colleges, asking the appointment of competent lecturers upon the diseases of the mouth and teeth as a means of good to the clientele of the physician.

7. Your committee regard the suggestions of the address touching professional ethics as furnishing matter for serious reflection, and ask a discussion and expression of opinion from this body with reference to this vexed and important question.

William Crenshaw,<br>

Thomas Fillebrown,<br>

A. H. Fuller,<br>

*Committee.*

Dr. Louis Jack then read his address, entitled "The Struggle for Professional Life, and its Relations with Practice." An abstract of the same follows:

He who has determined to enter into the practice of dentistry has commenced a course which in many of its aspects is attended with large and exacting responsibilities. He is to have in his keeping the preservation of an extremely essential portion of the human body, the derangements of which are far-reaching and fraught with general disturbances of the whole bodily economy. Therefore he requires the mastery of the principles and sciences which are related to the practice of medicine and surgery, added to the special knowledge of dental science, and also the skill in techniques and mechanics demanded to give practical efficiency. The student should keep always in view that it is a right principle to devote the strength of his mind and body to the requirements that scientific study and practical preparation impose upon every intelligent and rational mind, and that it is wrong and damaging to waste precious time in early manhood which should be devoted to study and research. The plans of the present system of the better colleges are intended to correct the disposition of the students to shirk duties, and to check the bad habits of overloading the mental stomach at the end of the term with a mass of indigestible pabulum which had better have been taken meal by meal. The brightest and the most useful men of all ages have been those who have taken up the battle by faithfully devoting themselves in their earlier years, in order to throw off the leaden weight of dulness and master their mental environments. The adjustment of plant, animal, or man to his environments is really what is meant by the familiar term, "The struggle for life." This struggle, which involves the survival of the fittest, is not as harsh as it may appear on the surface. It is upon all of us, and we cannot escape from it. With animals it is for bodily sustenance; with man it is as well for mental, moral, and spiritual growth.

The great purpose of life is the development of character. This is done by the toilsome rearing of truth upon truth, and cementing them into the mental constitution. Loyalty to one's profession may without impropriety be fittingly compared to patriotism, for to honor is to do everything which can render good to one's country, whether the service be of mind or the yielding up of life when it is required. We must be ministers and soldiers, as there may be need.

The foregoing does not appear on the surface to have much practical relation to the practice of dentistry; but is it not plain that the further elevation of dentistry depends upon the intellectual training of the younger men and the motive forces which animate all who engage in this work? We will often find that the result of the struggle which brings about the survival of the fittest will produce a broad and general advancement of the practice of dentistry, to the lasting and personal benefit of every honorable person engaged in the splendid and beneficent service which that profession is to mankind.

Dr. Jack, as chairman of Section III., reported on behalf of that committee. He stated that a marked feature of the period has been the development of the use of electricity in dental caries, for packing and finishing metal fillings, and in connection with therapeutic cataphoresis. Attention is called to a method of finishing gold fillings by hand-burnishers introduced by Dr. Libby. The combination of tin and amalgam beneath gold has been on the increase. Attention is called to the overwhelming increase of plastic filling-materials, many of which are very indifferent, but through extensive advertisement are praised into too wide an acceptance. The use of them raises the serious question, in view of the fact that most of the fillings are of short duration, whether dentistry as an art and our standing as a profession are not in danger of suffering a serious decline, and whether American dentistry is not falling into the conditions of practice which greatly prevail in Europe.

Dr. D. D. Smith, of Philadelphia, read a paper entitled, "The Office and Eccentricities of the Dental Pulp," of which an abstract follows:

Some months ago there was a talk before the Odontological Society of Pennsylvania on the office of the dental pulp. In the discussion that followed a wish was expressed that the subject-matter of that talk might be embodied in a paper to be read before the American Dental Association. In accordance with that wish the following has been gathered up for this occasion:

It is hardly to be expected that there can be presented to a body of dentists to-day anything specially new upon the office of the dental pulp. This organ and its functions have been so fully studied and are so thoroughly understood that it is doubtful if anything really new can be presented. It is possible, however, to get a new view of an old subject, as we might take a picture which has hung neglected for a long time because of an unsatisfactory light, and by rehanging it so change the light thrown upon it that it appears like a new picture, and there comes to us a new revelation of the artist's meaning. All we can hope to do at this time, in looking at the office of the dental pulp, is simply to present certain phenomena and facts in new groupings, and possibly to hang the picture in a better light. It is not the office of this paper to study the etiology of the teeth, or to look minutely at the formation of the teeth,—the enamel, dentine, and cementum. Neither is it to treat of the pulp action in the original formation of these structures. The intention is rather to speak of the part the pulp plays in the economy of tooth solidification, and the deductions therefrom. It is well known that the different formations which make up the body of the tooth receive light and nourishment from two distinct sources: one from the pulp, the other from the pericementum of the root. The full appreciation of this fact and the phenomena arising from it will greatly assist in establishing substantial and rational methods for saving the teeth. The pulp of the tooth is the central figure and the important factor in the tooth. To it is committed the care of the newly-erupted tooth, and its first work is to readjust, recalcify, consolidate, and strengthen and sustain the enamel and dentine. As a rule, the earlier a tooth is developed the more readily it yields to decay. The more time taken for calcification before eruption the greater the resistance to decay manifested on the part of the tooth. The young teeth are frequently erupted into environments which are most unfavorable to their development, and there begins at once a ceaseless contention between the forces acting externally to destroy it and the vital forces of the pulp to protect it from within. Just in proportion as the pulp is able to do its work of nourishing, consolidating, and maintaining the osseous parts will the crown at this period be protected from decay. Hence the emphasis that should be placed on keeping the pulps of young teeth in a condition of healthful activity.

The law of use governing tooth consolidation, widely known among patients, would do more to arrest the extensive decay now prevalent between eight and fifteen years of age than dentistry can ever hope to accomplish by mechanical .means. Too much importance has been and is attached to inheritance as determining the character of teeth. Use of the teeth in mastication, and thorough cleanliness will alone produce that exercise required by the pulp and peridenteum. The sixth-year molar, which erupts at a period when little thought or care is given to the teeth, is commonly the one over which the hardest battle for preservation is fought. The effort should be to save it, but with a living pulp.

Devitalization of the pulp at an early period of life carries with it a more or less rapid retrogressive change in the quality of the tooth-material. Fillings may prolong the existence of a tooth as such, but with the arrest of vitality in the tooth there is cessation of all vital sustaining action. The imperfectly calcified enamel and dentine built into the tooth are now in contact with devitalized connective tissue, which in the imperfectly consolidated tooth becomes a source of disintegration and assists in its destruction. The pulp is to the erupted tooth, whether temporary or permanent, the only source of life and sustenance, and it is of the first importance that it be maintained in a condition of health. All encroachments upon it through decay or manipulation should be carefully guarded against. So important is this that no effort should be spared to protect it from the progress of decay and injury. Its preservation in full activity means the deposition of better material, although parts of the crown may have been cut away. This opens up to us the whole subject of exposed pulps and the method of treatment, a domain into which I do not propose to enter. It has been said that the pulp is the only medium through which the dentine and enamel of a tooth are changed. It seems more than probable that the future may discover that the action is not confined to enamel and dentine alone, but that it extends into the cementum, exciting changes whereby true bone-structure is converted into a tissue resembling dentine more than cementum. Although a tooth may be fragile and imperfect at eruption, if you get it into a condition of use and cleanliness and the pulp is protected, the pulp will build up the tooth into an organ of good use. Instances of such cases are in the minds of all men of experience and observation. But we usually consider those cases exceptional rather than true expressions of the law of pulp action. Are not our literature and the teachings of the past and present wanting in that a position of supreme importance is given to operations on the external part of the tooth, while overlooking the important factor within the walls of the pulp-cavity? I would like just here to answer a question which was asked by one of the gentlemen in discussing dental education this morning. It was asked, What shall we do for the teeth of the poor? It is not strange that the question is asked, when we look abroad and see the condition of the mouths of a large percentage of the community. This question has come to every thinking mind. There is one work which, it seems to me, devolves upon the profession of dentistry, and which can be undertaken in its incipiency in this organization, and that is to emphasize the matter of education. Go into our public schools to day, and ninety-seven per cent. of all the scholars that you find there will be suffering with troubles of the teeth more or less important, much of which might be obviated by emphasizing just this single point, by forcing instruction into our public schools, by compelling the children to come to school with clean mouths, as they are required to come with clean hands. An influence might go out from dentistry into the community which shall reach to the lowest dregs by educating them as to the importance of their teeth and the importance of pulp action, so the tooth can be erupted into proper environments.

Does there ever come to a tooth a period in life when the pulp, with all the importance attaching to it in early life can be dispensed with? It is the contention of this paper that a pulp which has maintained a healthful existence in a tooth for twenty-five or thirty-five years has accomplished for that tooth all that it will or can do in the way of strengthening its osseous structure. The maximum of dentistry is reached with full maturity. Before this time the pulp forms part of the tooth; afterwards it can be dispensed with. If it remain within the tooth, there is an unmistakable restriction of its function and limiting of its activity, and sometimes there seems to be interference with the usefulness of the tooth. Having completed its work of consolidating the structures, it will sometimes commence to build in upon itself, circumscribing its boundaries, or depositing nodules of dentine or enamel, or pulp-stones, sometimes ossifying the whole coronal portion, and sometimes mummifying the entire substance by injecting into the tubuli an offensive coloring-matter, which baffles all efforts for its removal. Later in life we find teeth which have been decay-resisting for fifty or sixty years seemingly changing in structure, returning again to the conditions of childhood, as evinced by their yielding more readily to decay. Continuing on a little further, a few years more perhaps, it is not uncommon to find that decay has become extensive throughout the remaining teeth, along the gum margin particularly of the front teeth, where fillings have preserved them for twenty years or more; the teeth begin to deteriorate, and we find a change in the structure which is not imaginary, but positive. The tooth has softened. If refilled, after a short time they begin to darken around the surfaces. The fillings will no longer arrest decay as they have previously done. What has brought about this change? Is it not the pulp that previously rebuilt and consolidated the osseous structure of the crown,—the same pulp again at work, but now transforming the compact material which it built in that tooth into a condition wherein it yields much more readily to decay? What are the practical deductions from this action of the pulp? Suppose we take a tooth at the period of life when it is at its best, when the dentine and enamel are strongly consolidated, and destroy the pulp. What is the result? Only one result can follow. By no vital process can there be later in life any change of the compacted material of the dentine and enamel into any better or poorer formation. As the tooth is when the pulp is destroyed so it must remain, except as to those changes which take place through the gradual disintegration of the internal structures of all pulpless teeth. Have we injured the tooth in any material degree? What is the prognosis of a tooth with pulp destroyed at the period of maximum consolidation? With present methods of treatment, who would venture the prediction that a pulpless tooth would not continue in service to the end? If the pulp is so important at one period of life, and of little importance at another; if it is indeed a builder in early life and a source of disintegration in old age, shall we not revise our estimates of the value of the dental pulp?

One other point which this paper would simply hint at is this: It has been said that the future may demonstrate as a fact what is now a conjecture,—that the action of the pulp in rebuilding the osseous structures of the tooth is not confined in its operations to dentine and enamel, but extends an influence more or less potent into the territory of the cementum, depriving it of much of the characteristics of true bone-structure. If this be so, there comes a suggestion respecting some of the unexplained manifestations of pyorrhœa alveolaris. In this connection our first remark is, Pyorrhœa is seldom or never found in connection with young teeth. It seems to be a disease of adult life, generally of middle life. It is an affection not found in connection with soft teeth, nor with teeth much given to decay. So true is this, that in typical cases of pyorrhœa there is no decay in the teeth. It is never found in connection with devitalized teeth, where devitalization preceded the manifestation of the disease. In a mouth with extensive and uncontrollable pyorrhœa, it will be noticed that the disease is confined to the strong, well-formed teeth exempt from decay, not necessarily unfilled, but to teeth with living pulps. Our deductions therefore are these: If it should hereafter be found that the pulp sends a consolidating tendency or influence into the territory of the cementum, rendering it obnoxious to the pericementum because of too great consolidation, there will be found favorable conditions for the beginning of pyorrhœa, and we shall find in it the probable solution of the exemption of pulpless teeth from pyorrhœa. We shall also find why soft and young teeth are not affected, and why it is pre-eminently a disease associated with the hard, strong teeth of adult life. It is of vital importance that the cementum should be kept unimpaired and unchanged. It matters little what becomes of the pulp in a mature tooth. If the cementum is in a normal condition, it can be restored.

From the foregoing we must conclude that the dental pulp is an organ of the greatest importance up to the period of consolidation. After that, for a considerable period, as it would seem, it is of little service to the tooth, and later in life it may even become a source of injury and trouble. Of the gentlemen who have written of pyorrhœa no two seem to agree as to its etiology and methods of treatment. Age has not been considered a factor of any importance in connection with it, nor the character of teeth. It has been treated as a disease of local or constitutional origin, according to the view of the writer.

If what has been said will bear investigation, we must consider age, tooth-structure, and pulp-action, as well as local irritants and constitutional conditions, before it can be said that the origin of this troublesome concomitant of dental practice is satisfactorily defined.

Dr. J. D. Patterson, of Kansas City, then read a paper entitled "The Necessity of, and Methods for, Better Pulp Protection in filling Teeth," of which the following is an abstract:

In considering this subject, no reference will be made to pulps which have become exposed or in a pathological condition very near exposure, but to fairly deep-seated cavities in the teeth of patients under twenty-five years of age, which are usually filled without any endeavor to prevent pulp irritation, the operator believing that no considerable trouble will be possible, either from the presence of a filling with the property of conducting thermal changes, or from the irritant nature of filling-materials in common use. In this class of cases every observant practitioner has had his attention directed to the number of severe pathological conditions arising in after years from the death of the pulp. Where there is immediate or soon occurring trouble after a filling is inserted upon a nearly-exposed or pathological pulp, the removal of the filling and usual treatment bring immediate cure; but where the pulp-death comes from no continued irritation, the danger is greater. The patient's attention has not been called to the tooth until months or maybe years have elapsed, and when from an anæmic or diseased constitutional condition the dead remains of the pulp-tissue overcome the healthy function of the parts, the operator, be he as wise or as skilful in treatment as any, must often fold his hands, utterly unable to abate the alarming and rapid processes of destruction.

I will attempt to make a diagnosis of these cases, and show why they are so difficult to control. When the irritation to the pulp is slight, as it must be when it penetrates through the wall of dentine, the pulp responds without noticeable pain to the physiological action. If the putrid pulp contents are rapidly forced through the apical opening, the result is an immediate abscess; but in the slower process, from slight irritation, the poisonous matter infiltrates into the surrounding tissue in small quantities and intermittently, and Nature with its wonderful facility for destroying invading poisons, destroys the pathogenic bacteria and absorbs the detritus when it comes in small quantities. In this process, however, the investing tissues become weakened, and weekly or monthly the putrescent pulp matter again comes from the apical opening. Pathologists well know that an abscess voiding intermittently will seek new points of outlet, following more easily the healthy tissue than cicatrized territory. In this way each fresh invasion of pus will prejudice new territory, until irritation after irritation of the parts has placed the surrounding alveolar process and periosteal tissues, often to a distant point, in a weak and predisposed condition. There is no new power left in the tissues to withstand the attack, and at times great loss of process and teeth supervene through rapid inflammation and necrosis.

For pulp protection a variety of methods and materials have been adopted. Gutta-percha easily stands in first place, but it cannot be readily used except in deep cavities, where there is room for a good layer of cement over it, upon which to condense gold. Non cohesive gold in my practice is used for pulp protection almost to the exclusion of all other materials. Those who have been in practice many years cannot fail to notice that the old-time non-cohesive gold-fillings, which forty years ago were not universally used, upon removal were often found placed over pulps which were protected only by an extremely thin layer of dentine, and in this position had not caused any uneasiness from thermal changes. In these cases, if cohesive gold were used for refilling, the pulp was at once affected by hot and cold liquids and cold air, and even resulted in pulp-destruction. The philosophy of this difference in conducting power, between the two forms of gold, is not difficult to trace. In the non-cohesive filling, it is in no way a cohesive mass, cannot be welded together, and when a cavity is filled with layer after layer of this gold, the thermal shock is conducted along the layers and not down to the bottom of the cavity, for there is no intimate connection between the different particles of the different sheets. If you are annoyed with the effects of cold and heat in a cohesive gold filling, and will remove such filling, and without further treatment place over the pulp a pad of non-cohesive gold as here described, I can assure you that the trouble will at once disappear, and that afterwards you will treat all deep-seated cavities in this manner.

DISCUSSION.

*Dr. Friedrichs* (of Louisiana).—I shall only refer to the first paper read. I understood from that paper that a dead tooth, if the pulp be removed, is better preserved than one with the pulp alive. That certainly does not coincide with my experience. My experience in thirty-three or more years of practice is that the moment the pulp is destroyed the tooth is limited in regard to its duration; and also by observation I know that when the pulp is allowed to remain, as long as that tooth exists it will endure, for I have seen them worn down to the gums. The Almighty would not have placed us in such a position that after a certain period of time an organ that was built up should be destroyed before the period of our demise. He certainly intended that those teeth should last us as long as we lived, and we have plenty of cases where people have their teeth in good condition until they die. In regard to the cases of pyorrhœa alveolaris, I cannot see where the pulp has any action in that respect whatever. If such were the case, why do you find caries in mouths where only one tooth is affected and no others? Extract that tooth, and ten chances to one not another will be affected. Such cases have occurred to me in my own practice. If the pulp really were the cause of or would induce this condition, everybody would be affected with pyorrhœa alveolaris. Do you find that to be the case? Of course not. You find those lesions in the human body where one man may be affected with a certain thing, but it does not follow that the whole human race is affected. Where you have the pulp irritated, the usual trouble is the reflex condition of the pericementum. If the congestion remain in the pulp, pus is formed and the pulp is annihilated. There is no such thing in the pulp as an absorbent. There is a period of disease in dentistry, in regard to youth and old age. When senile decay steps in, as Dr. Atkinson said, it is want of proper nourishment, and those conditions then follow.

*Dr. McKellops* (of St. Louis, Mo.).—I think it is a pity that the very subject that every man here is interested in should go by without being discussed. These papers that have been read are very important, and they certainly should not be allowed to pass. More than half of the children who go to public schools are rich and able to take care of their teeth. They are not the poor children, as Dr. Smith represents. Go to your high schools, and you will find that the majority of those children are able to have their mouths attended to. I am interested in this matter of the pulp, and it is very important. When treating the sixth-year molar of a child you find a small portion decayed; you put in an amalgam or gold filling, and it is the most difficult thing in the world. You must have the confidence of that child, and learn to handle it with care, so you can accomplish some good result; and you do not do it by putting in any gold or amalgam filling, but by putting in carefully an oxyphosphate filling and watching it until the child is fourteen years of age; and I defy any man, if the filling is properly prepared and put in, to find a particle of decay there. When the child is fourteen years old, then put in your other filling, and put it in so no power on earth can affect it. Of course, some of them will wear out, but a platinum filling will last. I want the gentlemen of the profession to try this. You can come very near to matching the natural teeth, but if you put in gold the appearance is entirely different. It takes more care, of course, but it can be done. There has been no increase in that kind of work. There is no more platinum gold sold than there was years ago, but there is no better material that a man can use. With that material I can beat any inlay that was ever put in, and come so near to matching the teeth that it is almost impossible to notice it at a short distance.

In the charts that Dr. Patterson showed there must be a disease lurking in the system that causes all this trouble, syphilis, or something of that kind, that he has not studied out. You get up in the morning, and clean your mouth, and rub your finger along the glands, and you will get out a great quantity of mucus. So many people do not clean their teeth properly. I have used the fillings of gold for more than forty years, and I am a man who uses nothing but gold in my work without oxyphosphate. With brains, intelligence, and as bright minds as any society in the world, our men should not let the opportunity pass of discussing these papers. As a person begins to get old the nerves recede in the tooth; ninety per cent. of this trouble is caused by the secretions in the mouth, and we do not try to remedy it. I want Dr. Black to get some of those platinum fillings and to test them. If you take the oxychloride of zinc and put it in a tender tooth, you set up inflammation; but take a little iodoform and glycerin, and place it over the tender pulp, and a little asbestos paper over that, place in your oxyphosphate filling and let it set, and you can then put in any filling you want, and it will not irritate the tooth.

*Dr. Taft* (of Cincinnati, Ohio).—While hearing these papers read, the thought came to me that the first one should have come under the Section of Physiology, and that the second one should have come under the Section of Pathology, but that is not a matter of great importance. I simply want to emphasize a few thoughts that were expressed by the writer of the first paper in regard to the function and value of the pulps of teeth. The position he takes in regard to the use of this organ in the central portion of the tooth is correct, as every one knows, so far as the teeth of young persons are concerned; during the time of growth, development, perfection, and maturity of the tooth the pulp is a necessity. The tooth derives its nutritive supply from this source, and it goes on from its primary condition to its perfection (speaking of the permanent teeth); but what about it after this period? Is it of any value after the perfection of the tooth? Were it not of value in the economy, Nature would have made provision for its removal. Does the tooth need any nutrition after twenty-five years of age, after it has been entirely calcified? Can any one decide in any given case when the period of perfect calcification is reached? It is reached much earlier in some cases than in others. It is a fact patent to every close observer that the teeth in many instances do not seem to be complete in calcification at thirty or thirty-five years of age. How do we know? Up to this time they have remained in a comparatively deficient condition in this respect. They become more and more dense after this time. Does that increased solidification take place after the pulp is destroyed? Never. What are the constituents of dentine and enamel? Two general classes, organic and inorganic, vital and non-vital, the vital just as important an element as the non-vital. The vital must have supply and nutrition. When its life is taken away, deterioration at once begins, not in the broad sense manifesting disintegration at once, although that comes afterwards. The organic portion of the tooth is not nourished as when the pulp is living. Both the dentine and enamel receive their supply from the pulp of the tooth, and when this organ is destroyed this process of nutrition is also destroyed. It will be found that after a pulp is taken away, disintegration and breaking down of the tooth takes place without the ordinary process of decay, simply by a deterioration of the tooth-structure. That, of course, appears upon the organic material, and not the inorganic. Often we find a devitalized tooth with a portion of it broken off. The enamel far more readily breaks down, and disintegration occurs wherever a thin edge is left, or where there is an exposed edge or border, because of its weakened condition. That is true, also, of the dentine, because that has within it a much larger proportion of organic matter. I remember the case of an excellent tooth in a mouth in which all the teeth were excellent, except that the first permanent molar by decay had lost its pulp. There was a large cavity running through from front to back. Upon that tooth Dr. Atkinson many years ago performed an operation, filling up the pulp-chamber and building up the tooth. The statement was made that the tooth would last the lifetime of the patient, but it did not. It was a firm, solid tooth, as was its neighbors. That tooth lasted about eighteen years, and did good service, except that about twelve or thirteen years after it had been filled the edges of the enamel began to break away, and about eighteen years after it was filled the whole inner wall broke away. Examination revealed the fact that there was no ordinary decay. It was very slightly decalcified, but there had been such deterioration of the organic portion of the tooth that it was not able to withstand the force that was put on it in mastication and gave way. Within a year the other side broke off in the same manner, showing clearly that there was a deterioration of the structure or character of the tooth that was fatal to it.

I was very glad to hear the writer of the paper refer to the care of the teeth, especially in regard to mastication. If we say we know how to masticate properly, we sin against light and knowledge. We had better say we do not know. I have often asked dentists how much they urge their patients to masticate properly? Some of them say they never speak to their patients about it, and very rarely have I heard one say that he gave any special information or urged his patients to masticate thoroughly. It is not only the mastication, but the thorough insalivation, that is required. Those persons who masticate their food most thoroughly have the best teeth. They have the least dyspepsia and the best-nourished tissues in the body all through, and are better able to withstand all attacks of disease than those who do not masticate thoroughly. I know from observation that the majority do not masticate their food in anything like an adequate degree. I have noticed in this village a number of dentists, and I have observed that they take their meals in a few moments' time, the food not being thoroughly masticated nor thoroughly insalivated. I believe if the dentist can impress upon his patient the importance and the necessity of thorough mastication, that he has done one of the greatest services for his patient that is within his power. It is better than treating the diseases and conditions which we so frequently meet. It is hygiene of the mouth and the teeth, and it is for the benefit of the entire organization of the patient as well as of the teeth. The mother, the father, the nurse, and anybody in care of a child should notice it as early as three years of age, and teach it to masticate thoroughly and properly. The habit will stay with it through life, and prevent many of the ills and distresses that assail us.

*Dr. Truman* (of Philadelphia).—The discussion seems to have lost sight of the first paper. If the point brought out by Dr. Smith be true, it is an important factor for consideration. I am not prepared to believe that it is true. I think the discussion ought to lead in that direction. If I understood the paper, it was that the pulp from early life passes on to old age, and eventually degenerates, and resorption takes place. If this be a fact, it is an important one. I think all the evidence we have had in the past shows that there is an increasing density of the teeth up to the time that they are lost in advanced years, and that there never comes a time when there is resorption. This has been demonstrated recently by experiment. It has been theoretical heretofore. If I understood Dr. Black's series of papers in the *Dental Cosmos*, there is a gradual increase up to old age, and I would like to hear him speak on this point. I know from my own observation, microscopically and otherwise, that there is an increase in density in the structure of the tooth, but I know of no time when there is a resorption. There is at times a pathological condition apparent where the pulp destroys the tissue, but that is not what Dr. Smith means, if I understood him.

*Dr. Black.*—I would be glad to say a few words on this subject. The exact figures in regard to this matter any of you may study in the *Dental Cosmos* for May, 1895. It is a pretty long study, and will require some time to work itself out from the figures there so as to give a clear view. It is a fact that the teeth become more dense and their specific gravity becomes greater from youth to old age; but this difference is not great. It is a difference that requires the finest powers to demonstrate. The increase in strength is not very great. The difference between teeth is not very great, but it is certain. Follow those differences and you will see. Take the teeth of a young child, and you find average density; take the child in the teens and then in the twenties, and you find an increased density; then up to thirty there is slower increase, and from thirty to forty or forty-five the increase is very slight. From forty-five to sixty the increase in density is greater again. This is the way in which this has developed itself in my experimental work. I understand that Tomes is now repeating this work. I expect that he will practically substantiate these results, although not precisely, for no two sets of teeth taken from among one hundred persons will give the same results. The difference in density of the teeth of the same person is almost as great as the differences in the teeth of one hundred persons.

The influence of the pulp has been spoken of. Of course, the moment the pulp is dead the increase of density stops. This increase of density occurring in old age or in persons past middle age shows very plainly that the teeth require nutrition throughout life. In teeth that are worn down the pulp has receded, and the enamel has receded, and when it is worn away the strength of the dentine is impaired in proportion to the recession of the pulp. That wearing away of the teeth that we have come to regard as normal produces an abnormal condition of the tissues of the dentine. That tissue of the dentine has lost its vitality, fluids are admitted to the dentinal tubes, and that dentinal tissue becomes impaired and its strength is gradually lost in perfectly sound teeth. This is shown very clearly by experiments. Wherever we have a tooth that has lost its pulp and the vitality of the dentine is gone, and that tooth begins to show a discoloration, there we find that the strength of the dentine is impaired. When we come to test its strength, with the dynamometer, we find that the strength is impaired. We find a peculiar disposition of the enamel to chip off from the dentine. It is much more liable to be broken away, as my friend, Dr. Taft, has said. In the tooth that has its pulp and its proper nutrition, this parting from the dentine is not observed. This difference became very marked in this class of experimental work, all of which goes to show the value of the dental pulp, not simply in youth but throughout life. The breaking away that Professor Taft has spoken of and the causes of that breaking away seem to be well shown in this class of experimentation. The pulp is important, not only in youth but continually thereafter. As a person grows older its importance may be diminished, but it is still important, and it seems to me, after going over this class of experimentation, that if we can do anything to prevent the wearing down of the tooth, it becomes our duty to do so. Often we can build up here and there with the platinum gold that Dr. McKellops spoke of. We can build up the tooth with the harder material, thoroughly annealed and malleted, and we can make it much harder than hammered cast gold. We can make it stronger than the gold that we put through our rolling-mills and make into plates. It will bear more stress if we will give proper attention to the manipulation and the building of those fillings.

I do not know whether it is exactly under the subject of discussion, but I want to say a word about pulpless teeth, the effects upon the dentine of exposure of the pulp-chambers to the fluids of the mouth. If I do not follow it any further, I want to say that whenever we admit the fluids of the mouth to a pulp-chamber for twenty-four hours, we have injured that tooth for all time. It is now well known that when the pulp must be removed, if it be removed by the direct operation by the dentist, and the fluids of the mouth are not admitted at all, that the color of that tooth is maintained, if the filling be a perfect one. It is far more important that a filling be perfect in a pulpless tooth than in a tooth that has a living pulp. The deterioration and weakening of the dentine seems to be in some way connected with the admission of the fluids of the mouth to that dentine. The discoloration is dependent upon that, and wherever we get this discoloration I have found the weakened condition of the tissues of that organ. Wherever the tooth is bright, we find its strength good.

It is a mistake to suppose that light-colored teeth are weak teeth. They are not ; they are the strongest. Whenever we find them deteriorating in color, we find them deteriorating in strength, and the management that retains the color seems to retain the strength, whether it be in teeth that are wearing down or in pulpless teeth. That very admission of the fluids of the mouth to the tooth is the cause of the discoloration and the weakening of the tooth. During treatment, the greatest care should be exercised not only to prevent alveolar abscess afterwards, but to preserve the strength of the tissues of the tooth as well.

*Dr. Taft.*—Dr. Black says that when the tooth receives the fluids of the mouth, it changes color and is weakened; but while it retains its color, he says it retains its strength. I do not think he intended to say that it retains the strength it had while the pulp lived, or the strength it would have had if the pulp had remained alive in that tooth. There is a deterioration in every tooth when the pulp is destroyed, but far more marked in the cases mentioned than in those that retain their color. There is a deterioration in every tooth after the destruction of the pulp.

*Dr. Peirce.*—All papers that are presented to the Association that cannot be read here will be read by title and go into the hands of the Publication Committee.

*Dr. Crouse.*—This Association should adopt a different plan in regard to the section work, so that a section that takes two days shall not be crowded into one. It is not necessary for every section to report at each meeting. It ought to take two or three years for a section to prepare for a meeting of this Association, and two sections ought to take up the entire meeting. The report on Operative Dentistry that we are on now ought to occupy at least two days of the session. It is not necessary to hurry them through. They will improve by being kept until another year.

*Dr. Stephan* (of Cleveland).—The discussions of these papers have generally been short during the time I have been coming to these meetings. I come here to learn, and a discussion such as we have just had repays me for all my trouble in coming, and I hope we will have more of this, and not a crowding out of these papers.

*Dr. Crouse.*—I would move that all reports of the sections that we do not reach now shall not be read by title, but pass over until next year, and be taken up as part of our next year's work.

*Dr. Patterson.*—I am not in favor of that motion. It seems to me that it is not wise, and I think it should be voted down. If we have papers prepared, and they go over for a year, their value will be entirely lost. When one has spent a great deal of time in getting up a paper on a particular subject, the reputation which he has gained may be taken by some one else before the next year, and the work he has done counts for naught. If section work is done in the sections, if papers are read and passed upon by the sections themselves, and not slipped in, they ought to be published whether they are read or not.

*Dr. Crouse.*—Do you think it necessary to have all the sections report every year?

*Dr. Patterson.*—I have just said, let it go in the published report if the section passed on it. I do not want the matter crowded in, but I have not been in favor of adjourning when we were in the midst of an important discussion.

Dr. Crouse's motion laid on the table.

Adjourned.

(To be continued.)

